# *VviAPRT3* and *VviFSEX*: Two Genes Involved in Sex Specification Able to Distinguish Different Flower Types in *Vitis*

**DOI:** 10.3389/fpls.2017.00098

**Published:** 2017-01-31

**Authors:** João L. Coito, Miguel J. N. Ramos, Jorge Cunha, Helena G. Silva, Sara Amâncio, Maria M. R. Costa, Margarida Rocheta

**Affiliations:** ^1^Linking Landscape, Environment, Agriculture and Food, Instituto Superior de Agronomia, Universidade de LisboaLisboa, Portugal; ^2^Instituto Nacional de Investigação Agrária e VeterináriaTorres Vedras, Portugal; ^3^Instituto de Tecnologia Química e Biológica António Xavier, Universidade Nova de LisboaOeiras, Portugal; ^4^Biosystems and Integrative Sciences Institute, Plant Functional Biology Centre, University of MinhoBraga, Portugal

**Keywords:** *Vitis*, hermaphrodite, dioecious, flower, gene marker, sex

## Abstract

*Vitis vinifera vinifera* is a hermaphrodite subspecies, while its ancestor, *Vitis vinifera sylvestris*, is dioecious. We have identified two genes that together allow the discrimination between male, female and hermaphrodite *Vitis* plants. The sex locus region on chromosome 2 was screened resulting in the discovery of a new gene, *VviFSEX*. The same screening revealed another gene, *VviAPRT3*, located in the sex region, that be used as a sex marker. Both genes are good candidates to be involved in flower sex differentiation in grapevine. To assess their role in sex specification, spatial and temporal expression analysis was performed. The expression of *VviFSEX* is detected in petals, stamens and carpel primordia of all flower types, making its putative function unclear; however, female plants display a single allele for this gene, while male and hermaphrodites display two alleles. On the other hand, the specific expression of *VviAPRT3* in the carpel primordial of male plants suggests a possible role in the abortion of pistil structures. We propose a model to explain the carpel abortion in male flowers and the absence of stamen viability in female flowers. In addition, this work reinforces the presence of a sex locus on *Vitis* chromosome 2.

## Introduction

The cultivated grapevine (*Vitis vinifera* subs *vinifera*) is a self-pollinating hermaphrodite subspecies and it co-exists naturally with its ancestor (*Vitis vinifera* subs *sylvestris*) in many habitats throughout the Mediterranean area. *V. v. sylvestris* is dioecious with male plants producing flowers with erect stamens and fertile pollen, but also display a reduced pistil with no style or stigma (but with nectaries), whereas the female flowers have a perfect formed pistil (with style and stigma) but reflexed stamens with infertile pollen (Caporali et al., 2003). Nevertheless, at early developmental stages, male and female flowers are morphologically indistinguishable from a hermaphrodite flower, becoming unisexual only at later development stages, due to organ (Ramos et al., 2014).

In grapevine the sex locus responsible for sexual dimorphism has been identified in previous genetic mapping studies (Dalbó et al., 2000; Riaz et al., 2006; Marguerit et al., 2009), and is located close to the genetic marker *VviS3* (Dalbó et al., 2000) on chromosome 2^1^. The genetic marker *VviIB23*, which was used essentially in mapping populations, is also associated to the sex locus (Riaz et al., 2006). More recently, a region of 143 kb on chromosome 2 (12x_v0), between 4,907,434 and 5,050,616 bp, was identified as being responsible for sex specification in *V. v. vinifera* (Fechter et al., 2012). Although several genes in this region were good candidates to be involved in flower sex differentiation, only one of them, *ADENINE PHOSPHORIBOSYILTRANSFERASE* (*VviAPRT*), was identified as a marker able to discriminate female from male/hermaphrodite plants (Fechter et al., 2012). *VviAPRT* gene was located on chromosome 2 of the *V. v. vinifera* physical map based on the reference genome PN40024 8x version^2^, but is absent from the current *Vitis* genome annotation^1^ (12x_v2.1). Another study reported a 158 kb region, containing the previous 143 kb with linkage disequilibrium and genes exhibiting XY type polymorphism, such as *VviAPRT* (Picq et al., 2014). The latest annotation places this marker (VIT_200s1847g00010) on a set of unassigned scaffolds, referred in the *Vitis* database as “unknown chromosome.”

A chromosome 2 region (scaffold _154) was also identified as having homology with a section of the unknown chromosome (scaffold _233) in the 12x_v0 genome version (Fechter et al., 2012; Picq et al., 2014). It was hypothesized that this sequence on chromosome 2 corresponds to the female allele and the scaffold_233 sequence on the unknown chromosome corresponds to the hermaphrodite allele (Picq et al., 2014). Therefore, the reference genome PN40024 would be heterozygous regarding the sex locus (Picq et al., 2014).

*APRT* homologues in other species work as a key metabolic enzymes participating in cytokinin metabolism (Mok and Mok, 2001; Allen et al., 2002). In addition to auxins, cytokinins have been shown to be important contributors for flower sex specification in *Mercurialis* (Durand and Durand, 1991a,b). In *Arabidopsis thaliana, AtAPRT1* mutants develop male sterility due to atypical pollen formation (Moffatt and Somerville, 1988; Gaillard et al., 1998). In *V. v. sylvestris* male plants, the exogenous application of a synthetic cytokinin [6-benezylamino-9-(2-tetrahy- dropyranyl)-purine] induces the development of hermaphrodite flowers and the production of viable pollen and normal fruits (Negi and Olmo, 1966). Therefore, *VviAPRT* could be a possible candidate gene involved in sex specification in *V. v. sylvestris*, potentially through its influence in cytokinin metabolism.

This work is an attempt to understand the role of *VviAPRT3* gene in *Vitis* flower sex specification. Through *in situ* hybridization, in male, female and hermaphrodite flower tissues we determined the developmental stages and flower organs in which this gene is expressed. Additionally, a screening of the *V. v. vinifera* chromosome 2 allowed the identification of a new marker gene, VIT_202s0154g00200, (referred here as *VviFSEX*) that, in combination with *VviAPRT3*, allows the discrimination between male, female and hermaphrodite *V. vinifera* plants.

## Material and Methods

### Plant Sampling

**FIGURE 1:**
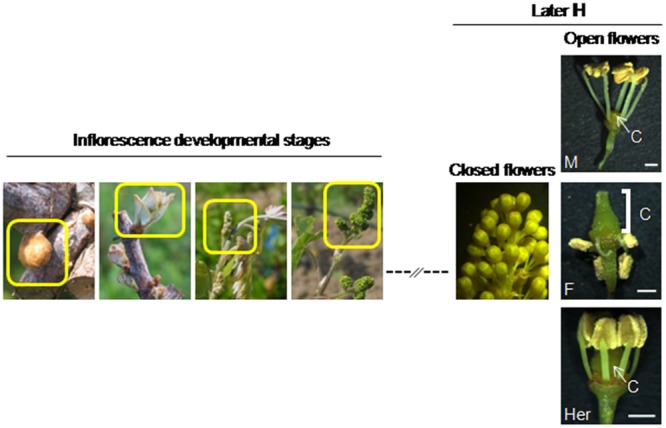
**Grapevine flower development stages.** Inflorescences of *Vitis* plants in flower development stages B, D, G and H (according to Baggiolini, 1952). After stage H is shown an inflorescence with closed flowers, similar in the three types of flower. Open flowers were dissected to obtain the carpel. Flower types are indistinguishable until the open flower stage when flower sexual organs are visible. Flowers of *V. v. sylvestris* male (M) plants display an incomplete carpel without style or stigma but with erect stamens. Flowers of *V. v. sylvestris* female (F) plants display a well developed carpel surrounded by reflected stamens, with infertile pollen. *V. v. vinifera*. Hermaphrodite (Her) flowers display a well-developed carpel and small erect stamens with fertile pollen. C, carpel. Scale bar = 500 μm.

For *in situ* hybridization and RT-qPCR, inflorescences at phenological developmental stages B, D, G, and H (Baggiolini, 1952) were collected from all *V. v. sylvestris* parental individuals of a collection, composed by 22 female (F) and 11 male (M) individuals. The same developmental stages were sampled from 12 hermaphrodites *V. v. vinifera* cv. Touriga Nacional (Her) (**Figure 1**) in Dois Portos (Lisbon district, Portugal) developmental stages B–D could not be used due to the woody nature of their tissues. However, at later stages (D and subsequent) stages the plants have several developmental flower stages within the same inflorescence.

Individual closed and open flowers (without flower cap) were collected from the three flower types at the later stage H, and carpels were dissected from open flowers and used in RT-qPCR (**Figure 1**). Additionally, leaves from *V. v. sylvestris* and *V. v. vinifera*, were collected for DNA extraction. Samples were collected during April (2014 and 2015).

### Nucleic Acids Extraction, cDNA Synthesis and Fragments Amplification

The DNA was extracted with the DNeasy^®^ Plant Mini Kit (Quiagen, USA) following the manufacturer’s instructions. Total RNA was extracted with the Spectrum^TM^Total RNA Kit (Sigma–Aldrich, Inc, USA) following the manufacturer’s instructions. The obtained DNA was stored at 4°C and the extracted RNA stored at -80°C between handling. The DNA and RNA concentration was determined using a Synergy HT Nanodrop system (Biotek, Germany), with the software Gen5^TM^ (Biotek, Germany).

cDNA from male, female and hermaphrodite inflorescences from the four flower developmental stages, as well as cDNA from closed flowers, open flowers and from carpels (**Figure 1**), was synthesized with the RETROscript^®^ kit (Ambion, Life Technologies, Spain), following the manufacturer’s protocol and amplified by PCR with specific primers (**Table 1**).

**Table 1:** Primers used for *VviAPRT3* and *VviFSEX* (VIT_202s0154g00200) gene amplification.

Target		Used	Orientation	Sequence (5′-3′)	Tm
Intron	*VviAPRT3*	Sex	Forward (F3)	TCTTTAGTATGAATGAATGTGC	55°C
		distinction	Reverse (R3)	AAACTCAGCCCTCCCTCA	55°C
Exon	*VviAPRT3*	RT-qPCR	Forward (F2)	GCATAGAAGCACGGGGTT	55°C
		*In situ*	Reverse (R1)	CATCAACTACCAAAGCACG	55°C
		Gene	Forward (F1)	AACCAGGGATTATGTTTCAAGA	55°C
		sequence	Reverse (R2)	CTTGCCATTCAATCGGTCACG	55°C
Exon	*VviFSEX*	RT-qPCR *In situ*	Forward	GCCCAGTATGTTATTGATTTAG	55°C
		Sex distinction	Reverse	TTCTTGGTGAGCAGATTATT	55°C

*Genomic *VviAPRT3* primers, targeting an intron covering the 2x repeat were used to discriminate between flower types. cDNA *VviAPRT3* primers, targeting four exons in total, were used in RT-qPCR analysis (F1 + R2), *in situ* probe synthesis (F1 + R2) and to determine the structure of the sequence (F1 + R1, F2 + R2, and F2 + R1, see Supplementary Figure 2). *VviFSEX* primers were used to discriminate between flower types, RT-qPCR analysis and *in situ* probe synthesis.*

To determine the most efficient cDNA concentration to be used for RT-qPCR, a serial of decimal dilutions was tested. Amplification reactions were performed with two biological samples per phenological stage in triplicates containing 5 μL of master mix (SsoFast_EvaGreen Supermix, Bio- Rad, USA), 0.4 μM of specific primers and 0.21 μg of cDNA in a 20 μL reaction, according to Ramos et al. (2014). The following program was applied: initial polymerase activation, 95°C, 2 min; 40 cycles at 95°C for 15 s (denaturation); 57°C for 30 s (annealing); 76°C for 30 s (extension) with a fluorescence reading at the end of each cycle. The run was completed with a melting curve analysis to confirm the amplification specificity. To confirm amplicon size, RT-qPCR products were run on 2.2% (w/v) agarose gel. RT-qPCR runs were performed, for each gene, with decimal dilutions series of a precisely calculated number of plasmid copies to create a calibration ruler. Cqs (threshold cycles) obtained from RNA samples was matched against the calibration ruler to estimate the number of transcripts. The absolute number of transcripts was calculated using the following formula: Number of transcripts = C × NA/M; where number of transcripts corresponds to the number of molecules μL^-1^ contained in the purified cDNA; C, concentration of the purified cDNA (g μL^-1^); M, the molecular weight of the cDNA gene fragment; NA, Avogadro’s number = 6.023 × 1023 molecules mole^-1^ (Whelan et al., 2003).

### Identification of Gene Sequences

The sequence of *ADENINE PHOSPHORIBOSYILTRANSFERASE* (*VviAPRT*) gene was first obtained from Genoscope 8x genome version (GSVIVT00007310001)^3^. Since a blast with this gene sequence against *Arabidopsis thaliana* database (TAIR^4^) revealed great homology with *AtAPRT3* (Fechter et al., 2012), from here on the gene will be referred to as *VviAPRT3*. Sequences from genes belonging to chromosome 2 in the previous identified 143 kb region (between 4,907,434 and 5,050,616 bp) (Fechter et al., 2012; Picq et al., 2014) and unknown chromosome were obtained from CRIBI Grape Database annotation 12x_v2.16^5^ (Supplementary Table 1). For clarity, the meaning of acronyms used for gene annotation is explained; for instance, VIT_202s0154g00200, referred here as *VviFSEX*, was identified on *Vitis vinifera* subs *vinifera* genome, on the sequencing 12x version v2.1 annotation (VIT_2), on chromosome 2 (02), scaffold s0154g and received the gene number 00200.

Genomic DNA and cDNA sequences of *VviAPRT3* and *VviFSEX* genes from the three flower types (male, female and hermaphrodite) were cloned into pGEM^®^ T-easy vector system (Promega, Leiden, The Netherlands) and transformed into *Escherichia coli* DH5α competent cells. After plasmid isolation (PureLink^TM^ Quick Plasmid Miniprep Kit, Invitrogen, Carlsband, CA, USA) and sequencing, the sequences were aligned using Clustal Omega (Sievers et al., 2011; McWilliam et al., 2013; Li et al., 2015).

### RNA *In situ* Hybridization

Plant tissue fixation, clearing, and *in situ* hybridization experiments were performed as previously described (Coen et al., 1990) with the following modifications: for the probe synthesis the cDNA of *VviAPRT3* and *VviFSEX* (VIT_202s0154g00200) were cloned into the pGEM^®^ T-easy vector system and amplified by PCR with the M13 forward/reverse primers and specific forward/reverse primers (**Table 1**). The PCR product was purified using the MinElute PCR Purification Kit (QIAGEN, Valencia, CA, USA), according to the manufacturer’s instructions and used as template for the riboprobe synthesis, which was carried with SP6 and T7 RNA polymerase to obtain the sense and antisense strand. The paraffin embedded material was sectioned at 7 μm and the tissue slices mounted with distilled water. Images were captured with a fluorescence microscope (Wild Leitz, Laborlux S) with an AxioCam HRM (Zeiss).

### RT-qPCR Statistic Analysis

For the statistical analysis, expression values of *VviAPRT3* and *VviFSEX* were transformed into log_2_ and tested through ANOVA using the program Graphpad Prism 5 (GraphPad Software, Inc.).

The samples with a *p*-value of the ANOVA lower than 0.05 were submitted to an additional Tukey test. The statistically significant differences were accepted when Tukey’s test *p*-value was lower than 0.05.

## Results and Discussion

*Vitis vinifera* species display dioecious and hermaphrodite sexual systems, in which three types of flowers are observed: males and females in *V. v. sylvestris* and hermaphrodites in the cultivated subspecies *V. v. vinifera*. Male flowers are characterized by having long erect stamens and a reduced carpel without style or stigma, but with nectaries and ovaries (**Figure 1**). Female flowers have a complete carpel with style and stigma but short and reflex stamens (**Figure 1**) with infertile pollen (Caporali et al., 2003; Gallardo et al., 2009). The hermaphrodite flower displays functional male and female organs. The pistil is perfectly formed and fully functional with style, stigma and ovaries, and the stamens, although shorter than the male ones, are erect and produce viable pollen (**Figure 1**) (Carmona et al., 2008; Ramos et al., 2014; Valleau, 1916).

### *VviFSEX:* a New Female Sex Marker

**FIGURE 2:**
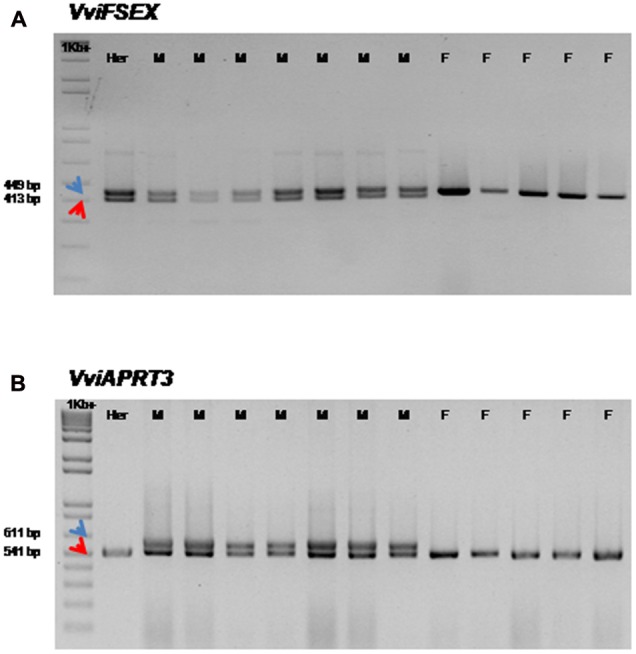
**Genomic amplification of *VviFSEX* and V*viAPRT3* genes in a *Vitis* population. (A)** *VviFSEX* male plants (M) and the hermaphrodite (Her) display two fragments of 449 bp (blue arrow) and 413 bp (red arrow). Female (F) plants display a 449 bp fragment. **(B)** *VviAPRT3* in male plants (M) display two fragments, one of 611 bp (blue arrow) and another of 541 bp (red arrow). The hermaphrodite (Her) and the female plants (F) display a 542 bp fragment. These amplifications were performed in all 45 plants (22 female; 11 male and 12 hermaphrodites *V. v. vinifera* cv. Touriga Nacional). Fragments size was determined by sequencing. 1 Kb^+^, genetic molecular marker.

Previous work suggests the existence of two alleles on chromosome 2 of *V. v. vinifera* due to the high homology of this region with one of the unknown chromosome (Picq et al., 2014). Due to the putative existence of two alleles, we have analyzed all the genes present in the 143 kb region that are assigned to the scaffold_154 of chromosome 2 genome version 12x_v0 (VIT_202s0154gxxxxx) (Supplementary Table 1). The analysis showed eleven genes with high homology with genes present in scaffold_233 of the unknown chromosome (VIT_200s0233gxxxxx) (Supplementary Table 1). A pairwise alignment between homologous genes in both scaffolds revealed some sequence differences that were used to design PCR primers able to discriminate these genomic loci and, potentially, providing molecular markers for plant sex identity. Subsequently, these primer pairs were used to genotype 22 female, 11 male and 12 hermaphrodite plants. Based on the presence/absence of the amplified PCR products and their respective sizes, we noticed that only VIT_202s0154g00200 (*VviFSEX*) displays the differences predicted *in silico*. Two fragments with 449 and 413 bp were amplified from the DNA of male and hermaphrodite plants while a single fragment of 449 bp was amplified from female plants (**Figure 2A**). This genotyping approach allows the discrimination of female from male and hermaphrodite plants, and thus the *VviFSEX* gene sequence may be used as a genetic marker for female plants. The exact size of PCR products amplified from male, female and hermaphrodite was determined by sequencing analysis. This analysis showed that the 449 bp fragment present in all the flower types, despite being homologous to the 413 bp fragment of male and hermaphrodite individuals, contains an extra 36 bp region (Supplementary Figure 1). The sequence with the additional 36 bp may correspond to an alternative allele of this gene, being homozygous in female plants and heterozygous in male and hermaphrodite plants. Picq et al. (2014) had already suggested that a region of unknown chromosome homologous to the 143 kb region of chromosome 2 (Fechter et al., 2012) may correspond to different alleles of the same genes. In the present work we also identified several genes in the same scaffold of unknown chromosome with great homology to genes found on chromosome 2 (Supplementary Table 1). The comparison between the genomic sequences revealed some differences that suggest the existence of possible alleles.

It is important to note that the existence of an unknown chromosome in the reference genome (PN40024) indicates that the annotation is not concluded and it requires an additional effort to improve gene chromosome assignment (Jaillon et al., 2007).

#### Spatial and Temporal Expression of *VviFSEX*

**FIGURE 3:**
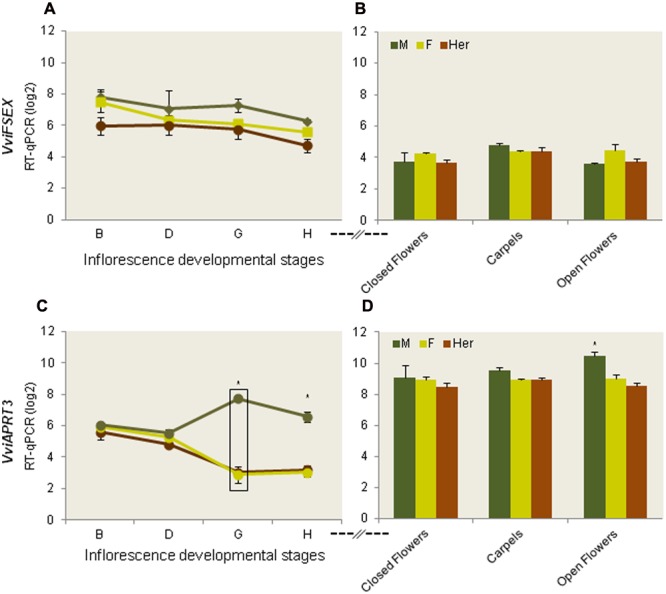
**Absolute quantification by RT-qPCR of *VviFSEX* and *VviAPRT3* transcripts during flower development.** Expression of *VviFSEX* **(A,B)** and *VviAPRT3* **(C,D)** genes in all three flower types in four development stages **(A,C)** and in closed flowers, mature flowers (open flowers) and carpels **(B,D)**. M, male (dark green); F, female (light green); Her, hermaphrodite (brown) in log_2_ (absolute quantification). Error bars represent standard error. Significant differences were tested through ANOVA and the samples with a *p*-value lower than 0.05 were submitted to an additional Tukey test. Differences were accepted when Tukey’s test *p*-value was lower than 0.05. Statistical differences were found in G developmental stage when comparing with the previous stage, in all flower types and are represented by a square box. (^∗^) represents significant differences (*p* < 0.05) when comparing the developmental stages between the three flower types.

After establishing *VviFSEX* as a marker gene for flower sex in grapevine, its spatial and temporal expression was analyzed during the development of male, female and hermaphrodite flowers. We cloned and sequenced the cDNAs from the three flower types and we found that they are identical (data not shown). RT-qPCR analysis of *VviFSEX* in flower developmental stages B, D, G, and H showed constant abundance of transcripts in all the developmental stages with no significant variation between the three flower types (**Figure 3A**). All the flower types follow a bisexual development pattern during the three early stages of floral development sampled (B, D, and G), but unisexuality arises by organ abortion in late stage H, when the maturity of all flower organs takes place.

**FIGURE 4:**
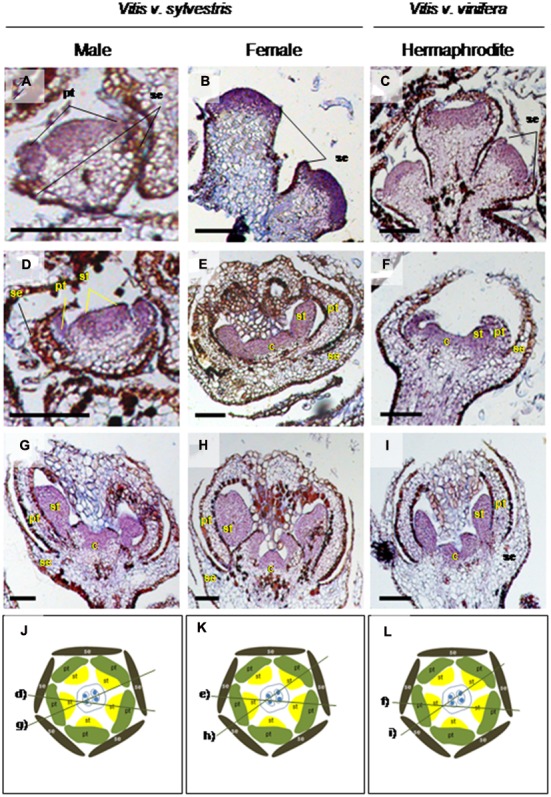
***In situ* hybridization of the *VviFSEX* gene in several flower developmental stages in three *Vitis* flower types.** The transcript of *VviFSEX* gene is present in early flower developmental stages in male **(A)**, female **(B)** and hermaphrodite **(C)** plants when the floral primordia display emerging sepals and/or petals. When stamens are visible *VviFSEX* is expressed in petals, stamens and carpel region, throughout the three flower types **(D–F)**. Later, the gene transcript remains in stamens and in the carpel but seems to be absent from petals **(G–I)**. Cutting planes are represented in **(J–L)** with the respective slide section outlined. Cuts were performed with 7 μm thick. Flowers structures: se, sepals; pt, petals; st, stamens; c, carpel. Scale bar = 100 μm.

The expression of *VviFSEX* was also analyzed in samples of later flowering stages ((closed flowers, open flowers and carpels (dissected from open flowers)) by RT-qPCR (**Figure 1**). We found that the expression level of *VviFSEX* is similar between female, male and hermaphrodite samples (**Figure 3B**). To evaluate the spatial expression pattern of *VviFSEX* in the different flower types, the transcript accumulation was analyzed in male, female and hermaphrodite flower sections by i*n situ* hybridization. *VviFSEX* transcript was detected at early flower developmental stages in petal primordia and flower meristems in the three flower types (**Figures 4A–C**). When stamen primordia become evident, *VviFSEX* expression is observed in petals, stamens and in the carpel in all flower types (**Figures 4D–F**). Later in development, the expression remains in stamens and carpels but is no longer present in the petals (**Figures 4G–I**). The abundance and localization of *VviFSEX* transcripts is similar in the different flowers types of *V. vinifera* species. The analysis of *VviFSEX* protein sequence by the HMMER web server^6^ did not reveal significant homology with known proteins. However, we do not rule out a putative role of this gene in sex specification processes of grapevine due to its strong specific expression in the reproductive structures at later developmental stages. In fact, *VviFSEX* transcript is present in early stages in whorl 2, 3 and 4 but later in development it is retained in whorl 3 and 4 and excluded from whorl 2 (**Figure 4**). This may indicate that *VviFSEX* acts redundantly to form petals in early stages and stamens and carpels in late stages (**Figure 4**).

### *VviAPRT3*: a Maker for Male Plants

According to Fechter et al. (2012), the *VviAPRT3* gene was located in a 143 kb region and it might have a role in flower sex specification. These authors used the second intron of *VviAPRT3* as a marker able to differentiate female plants of several different *Vitis* species, including *V. v. vinifera* and *V. v. sylvestris* subspecies (Fechter et al., 2012). However, it was not possible to amplify the *VviAPRT* fragment previously described Fechter et al. (2012) in the plant material used in the current work, since the primers or the gene sequences were not provided. The *VviAPRT3* marker was located on the chromosome 2 of the reference genome between 4,986,517 bp and 5,025,265 bp (Fechter et al., 2012). In the 8x genome reference sequence on Genoscope database^7^, *VviAPRT3* was located 200 kb downstream of the reported location (GSVIVT00007310001). In fact, the sex region of 143 kb in the 12x_v2.1 was located roughly 100 kb downstream in the 8x version of the reference genome, although most of the genes were kept together (Supplementary Table 2). However, in the 12x_v0 and v2.1 versions, *VviAPRT3* was allocated to the unknown chromosome (VIT_200s1847g00010) (Supplementary Table 2) with a different genomic structure (Supplementary Figure 2). In *Vitis*, the expression levels of *VviAPRT3* was first reported in the transcriptome assembly of different stages of flower development (Ramos et al., 2014). The transcriptomic data revealed that *VviAPRT*3 gene is differentially expressed in male, female and hermaphrodite plants. The putative involvement of this gene in grapevine sex specification and the disagreement between annotation and sequencing versions led us to further investigate its genomic structure. To determine which annotation version stands correct regarding *VviAPRT3* structure, several primers were designed to amplify *VviAPRT3* (Supplementary Figure 2). PCR amplification of cDNA with primers for the first and last exons (F1 + R2) originated a fragment of 443 bp in the three flower types (Supplementary Figure 2). cDNA sequencing revealed no differences between the fragments amplified from the different flower types (data not show). Amplification with primers for the third and fourth exons (F2 + R1) resulted in a fragment of 689 bp, while the amplification with primers for the fourth and sixth exons (F2 + R2) resulted in a fragment of about 3,000 bp (Supplementary Figure 2). By using these specific primer combinations (Supplementary Figure 2), and sequencing the corresponding amplified fragments, we concluded that *VviAPRT3* genomic structure, with six exons, five introns and a 2x repeat in the second intron, was accurately represented in the 8x genome version. Therefore, the *VviAPRT3* sequence subject of this work and *VviAPRT* from Fechter et al. (2012) are most likely the same gene (Supplementary Table 2). Fechter et al. (2012) reported that *VviAPRT3* was able to discriminate female flowers from the other flower types based on a 2x repeat present in the second intron in male and hermaphrodite plants. Thus, we designed primers flanking the 2x repeat in the second intron (F3 + R3) of *VviAPRT3* (**Table 1**; Supplementary Figure 2) that amplify the tandem repeat region in the three flower types. Instead of distinguishing female from other plants, we were able to distinguish male from female and hermaphrodite plants. These plants displayed a fragment of 541 bp (**Figure 2B**), while male plants showed an additional fragment with 611 bp (**Figure 2B**). By sequencing the 541 bp fragments from each flower type it was possible to demonstrate that their genomic sequences are similar (Supplementary Figure 3). The fragment of 611 bp found solely in the male plants results from an insertion of 70 bp that is absent in female and hermaphrodite plants (Supplementary Figure 3).

#### *VviAPRT3*: a Putative Player in *Vitis* Sex Specification

**FIGURE 5:**
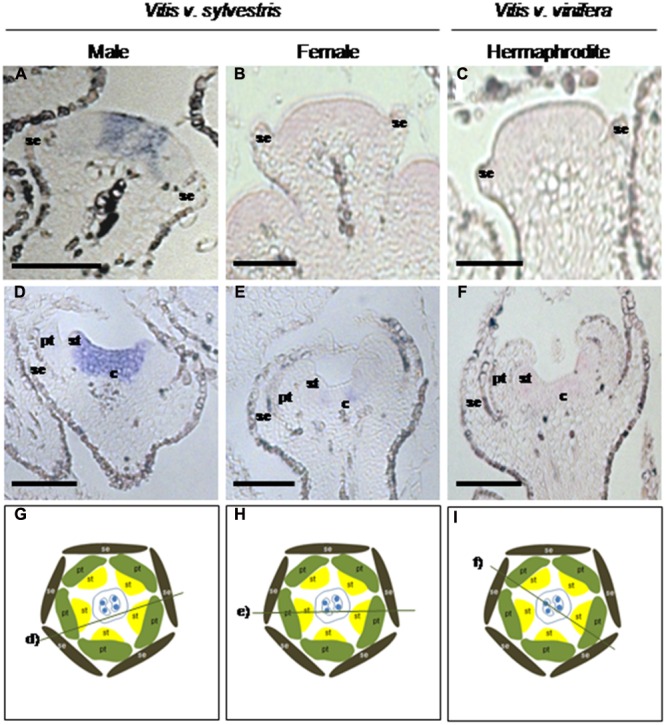
**Analysis of *VviAPRT3* expression during early flower development by *in situ* hybridization in three *Vitis* flower types.** Expression of *VviAPRT3* in early developmental stages in male **(A,D)**, female **(B,E)** and hermaphrodite **(C,F)** flowers. *VviAPRT3* expression is visible in male flowers (in blue/pink) in the center of flower meristem where the carpel will emerge **(A)**. When stamen primordia become distinguishable, the expression expands to the internal side of the stamens **(D)**. Expression of *VviAPRT3* could not be detected in female and hermaphrodite flowers in early flower development **(C,D)**, although it becomes visible when stamens begin to emerge **(E,F)**. Cutting planes are represented in **(G–I)** with the respective section outlined. Cuts were performed with 7 μm thick. Flowers structures: se, sepals; pt, petals; st, stamens; c, carpel. Scale bar = 100 μm.

To determine the spatio-temporal expression of *VviAPRT3*, the accumulation of transcripts was analysed in the four stages of flower development used in this work (B, D, G, and H) in male, female and hermaphrodite flowers of grapevine, by RT-qPCR and *in situ* hybridization (**Figures 3** and **5**).

The expression pattern of *VviAPRT3*, previously found in the transcriptomic analysis of grapevine flowers (Ramos et al., 2014), was confirmed by RT-qPCR. *VviAPRT3* gene has similar levels of expression at very early stages of flower development in all flower types (stage B and D). At G and H stages, the expression decreases in female and hermaphrodite flowers, whereas in male flowers the expression increases considerably (**Figure 3C**). Additionally, the expression of *VviAPRT3* in closed flowers, open flowers and in carpels is comparatively similar (**Figures 1** and **3D**) but a considerably higher expression in male open flowers is still evident (**Figure 3D**). The increase in *VviAPRT3* expression in stage G (early development of reproductive structures) of male flowers when compared to female and hermaphrodite, suggests that this gene may play a role in sex specification at late flowering stages in grapevine.

**FIGURE 6:**
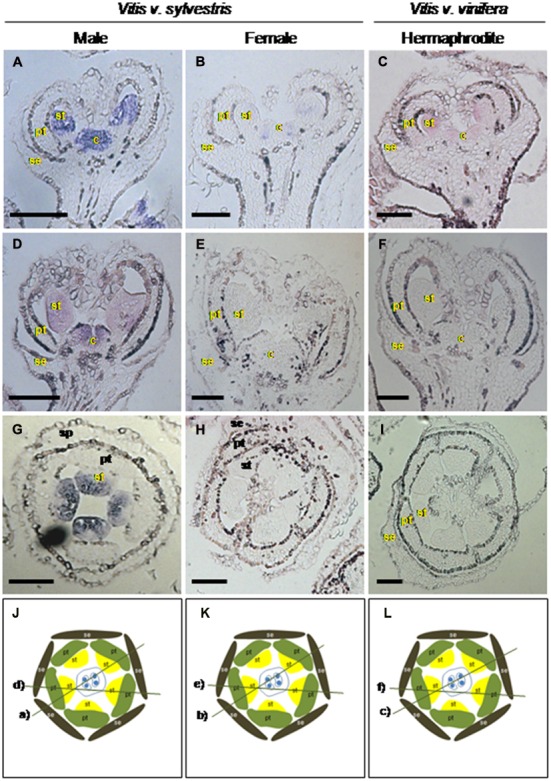
**Analysis of *VviAPRT3* expression during late flower development by *in situ* hybridization in three *Vitis* flower types.** Expression of *VviAPRT3* in late developmental stages of male **(A,D,G)**, female **(B,E,H)** and hermaphrodite **(C,F,I)** flowers. *VviAPRT3* expression is visible (in blue/pink) in carpels and stamens of male plants **(A,D)**. *VviAPRT3* expression in the stamens is confined to the internal side **(G)**. When petals start to enclose the sepals, *VviAPRT3* expression is almost undetectable in female and hermaphrodite flowers (**B,C**, respectively) but completely disappears in later stages (**E,H**: female; **F,I**: hermaphrodite). Longitudinal sections **(A–F)**; Transversal sections **(G–I)**. The cut plane of transversal sections is on bud top above the carpel. Cutting planes are represented in **(J–L)** with the respective longitudinal section outlined. Cuts were performed with 7 μm thick. Flowers structures: se, sepals; pt, petals; st, stamens; c, carpel. Scale bar = 100 μm.

The spatio-temporal analysis of *VviAPRT3* expression by i*n situ* hybridization during early flower developmental stages, when floral meristems emerge, showed that this gene is expressed in the center of the male flower meristem, where the carpel primordia would arise (**Figure 5A**). The transcript accumulation of *VviAPRT3* could not be detected in similar developmental stages of hermaphrodite and female flowers (**Figures 5B,C**). When male stamens primordia start to emerge, the expression of *VviAPRT3* spans from the center of the flower to the inner sides of stamens (**Figure 5D**). At the same stage, in female and hermaphrodite flowers, the expression is very faint (**Figures 5E,F**), which is in agreement with the RT-qPCR results (**Figures 3C,D**). Later in development, when petals start to enclose the stamens and carpels, it is possible to visualize *VviAPRT3* expression in carpel and stamens of male flowers (**Figure 6A**). At a similar stage, the mRNA of *VviAPRT3* is almost undetectable in female and hermaphrodite flowers (**Figures 6B,C**). When sepals fully encapsulate the stamens and the carpels, *VviAPRT3* expression is maintained in male stamens and carpels (**Figure 6D**). However, no detection of *VviAPRT3* transcripts was observed in any flower organs of the female and hermaphrodite flowers, at the same stages (**Figures 6E,F**). Transversal sections of flowers show unambiguously the presence of *VviAPRT3* transcript in male stamens and its absence in female and hermaphrodite flowers (**Figures 6G–I**).

The apparent differences between RT-qPCR and *in situ* hybridization results at early stages B and D are due to the type of sample used and the nature of the technique. Each inflorescence phenological stage (Baggiolini, 1952) encloses flower meristems at different developmental stages. The RT-qPCR was performed to evaluate the temporal expression considering the inflorescence phenological stage. On the other hand, the *in situ* hybridization allows the observation of spatial and temporal gene expression considering the developmental stage of a specific flower meristem.

**FIGURE 7:**
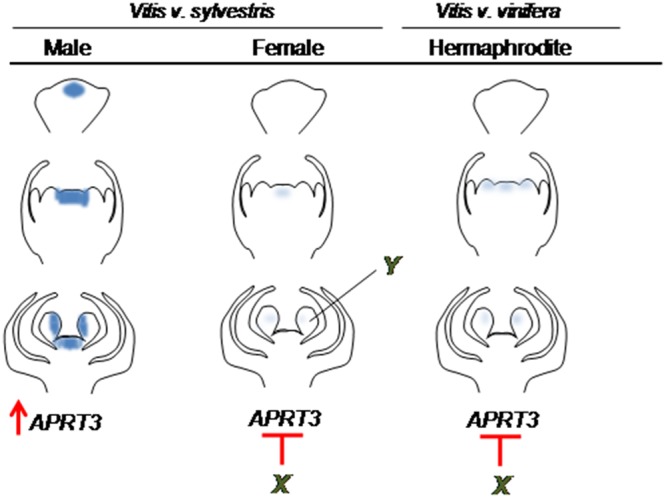
**Schematic representation of *VviAPRT3* expression in three developmental stages of *Vitis* flower development.** *VviAPRT3* is highly expressed in male plants in the 3rd and 4th whorl with a possible role in the arrest of carpel formation. Its absence in female and hermaphrodite flowers point for the presence of a gene (X) that inhibit *VviAPRT3* in these flowers allowing the correct carpel formation. Additionally, the correct stamens development in male and hermaphrodite flowers and the reflexed stamens in female leads us to anticipate the possibility of another gene acting in the 3rd whorl that in female flowers make reflexed stamens (with unviable pollen).

In *Arabidopsis thaliana* an homologous gene of *VviAPRT3, AtAPRT1*, is involved in reproductive development by playing an essential role during pollen development (Moffatt and Somerville, 1988). However, the lack of *VviAPRT3* expression in the stamens of hermaphrodite flowers at late stages of development, suggests a different role in the *Vitis* genus. *VviAPRT3* expression in the carpel primordia of male flowers hints a specific role for their development, since female and hermaphrodite flowers have no detectable *VviAPRT3* transcripts. The expression of *VviAPRT3* in the central whorl of male flowers may interfere with the normal development of the pistil and influence its later arrest. In *Arabidopsis thaliana, AtAPRT1* codes for a key metabolic enzyme that plays a role in maintaining cytokinin homeostasis (Zhang et al., 2013). In *Vitis*, hormones can modify flower sex identity, with cytokinins playing a major role in the process (Negi and Olmo, 1966, 1971; Zhang et al., 2013). Exogenous application of this hormone in *V. v. sylvestris* converts male flowers to hermaphrodites (Negi and Olmo, 1971), bypassing the genetic regulatory mechanisms that suppress pistil development (Iizuka and Hashizume, 1968). Thus, high levels of cytokinins might be essential for carpel development. In male flowers of *V. v. sylvestris*, the *VviAPRT3* enzyme activity may be promoting the inactivation of cytokinins, which may lead to carpel abortion. Considering our results, we propose a model where in female and hermaphrodite flowers the activity of a putative regulatory gene/element may block the *VviAPRT3* transcription, and thus the female organ can correctly develop (**Figure 7**). Also, its absence in stamens of hermaphrodite and female flowers, suggests that *VviAPRT3* is not involved in the correct formation of male organs (**Figure 7**). However, we do not rule out the hypothesis that the over expression of *VviAPRT3* in male plants could have a dual role: the enhancement of male traits (Gallardo et al., 2009) and a participation in carpel arrest. To explain the differential expression in stamens of the three flower types, we propose the action of another gene in the third whorl of female flowers with a role in stamens deflection and consequently the formation of unviable pollen (**Figure 7**).

## Conclusion

In this work, we found two genes that when analyzed simultaneously can be a valuable resource in a breeding program, since they allow the distinction between *V. vinifera* individuals with different flower types, male, female or hermaphrodite. Although the molecular pathways leading to dioecy in grapevine are yet to be clarified, the information provided by this work suggests that *VviAPRT3* may be a player in male sex specification. The presence of *VviAPRT3* transcripts in the carpel primordia of male plants indicates a possible function in the arrest of this organ in male flowers. Its function in the third whorl of male flowers is unclear; however, the absence of its expression in the female and hermaphrodite flowers rules out a role in anther development, since its presence would be necessary in hermaphrodite flowers for proper stamen development. Additionally, the phenotype of female flowers suggests the activity of another gene that causes the abnormal stamen development of stamens.

## Author Contributions

JC, MR, and MC were involved in experimental design and interpretation of data; JC establish *V. v. sylvestris* collection and collected morphologic data; JC and HS performed experiments; JC wrote the manuscript; MR, MNR, HS, MC and SA reviewed and edited the manuscript; MR supervised experiments. All authors contributed to editing and approving the final version of the manuscript.

## Conflict of Interest Statement

The authors declare that the research was conducted in the absence of any commercial or financial relationships that could be construed as a potential conflict of interest.

The reviewer MC and handling Editor declared their shared affiliation, and the handling Editor states that the process nevertheless met the standards of a fair and objective review.

## Footnotes

1 http://genomes.cribi.unipd.it/grape/

2 http://www.genoscope.cns.fr/externe/GenomeBrowser/Vitis/entry_ggb.html

3 http://www.genoscope.cns.fr/externe/GenomeBrowser/Vitis/

4 https://www.arabidopsis.org/

5 http://genomes.cribi.unipd.it/gb2/gbrowse/public/vitis_vinifera_v2/

6 http://hmmer.org/

7 http://www.genoscope.cns.fr/externe/GenomeBrowser/Vitis/
