# Supplementary material for: VviAPRT3 and VviFSEX: Two Genes Involved in Sex Specification Able to Distinguish Different Flower Types in Vitis
Source: Front Plant Sci. 2017 Jan 31;8:98. doi: 10.3389/fpls.2017.00098 (PMC5281589; doi:10.3389/fpls.2017.00098)
Supplement: Supplementary file 1 [file Presentation_1.PDF]

Supplementary Material

VviAPRT3 and *VviFSEX*: two genes involved in sex specification able to distinguish different flower types in *Vitis*

João Lucas Coito[1], Miguel Jesus Nunes Ramos[1], Jorge Cunha[2,3], Helena Gomes Silva[4], Sara Amâncio[1], Maria Manuela Ribeiro Costa[4], Margarida Rocheta[1*]

[1]Universidade de Lisboa, Instituto Superior de Agronomia, LEAF, Linking Landscape, Environment, Agriculture and Food, Tapada da Ajuda, 1359-017 Lisboa, Portugal

[2]Instituto Nacional de Investigação Agrária e Veterinária, Quinta d'Almoinha, 2565-191 Dois Portos, Portugal

[3]ITQB, Universidade Nova de Lisboa, 2780-157 Oeiras, Portugal

[4]Biosystems and Integrative Sciences Institute (BioISI), Plant Functional Biology Centre, University of Minho, Campus de Gualtar, 4710-057 Braga, Portugal

* Corresponding Author:

rocheta@isa.ulisboa.pt

```
M_413        GCCCAGTATGTTATTGATTTAGAAGAATCAACAGAAAAAGGATCAGATGAGGATACTCAA
Her_413      GCCCAGTATGTTATTGATTTAGAAGAATCAACAGAAAAAGGATCAGATGAGGATACTCAA
M_449        GCCCAGTATGTTATTGATTTAGAAGAATCAACAGAAAAAGGATCAGATGAGGATACTCAA
F_449        GCCCAGTATGTTATTGATTTAGAAGAATCAACAGAAAAAGGATCAGATGAGGATACTCAA
Her_449      GCCCAGTATGTTATTGATTTAGAAGAATCAACAGAAAAAGGATCAGATGAGGATACTCAA
             ************************************************************

M_413        ACTGTGTCCCCTCTCATCTTTGCTGGCCTGACCCCTTCTTCAGGAGACAAGCATGAGCTG
Her_413      ACTGTGTCCCCTCTCATCTTTGCTGGCCTGACCCCTTCTTCAGGAGACAAGCATGAGCTG
M_449        ACTGTGTCCCCTCTCATCTTTGCTGGCCTGACCCCTTCTTCAGGAGACAAGCATGAGCTG
F_449        ACTGTGTCCCCTCTCATCTTTGCTGGCCTGACCCCTTCTTCAGGAGACAAGCATGAGCTG
Her_449      ACTGTGTCCCCTCTCATCTTTGCTGGCCTGACCCCTTCTTCAGGAGACAAGCATGAGCTG
             ************************************************************

M_413        CAAGCTTCTGTTTTATCTAATCTGGCCACCTCCAAAAGTGGAAAGAAAGATCCAGAAGAT
Her_413      CAAGCTTCTGTTTTATCTAATCTGGCCACCTCCAAAAGTGGAAAGAAAGATCCAGAAGAT
M_449        CAAGCTTCTGTTTTATCTAATCTGGCCACCTCCAAAAGTGGAAAGAAAGATCCAGAAGAT
F_449        CAAGCTTCTGTTTTATCTAATCTGGCCACCTCCAAAAGTGGAAAGAAAGATCCAGAAGAT
Her_449      CAAGCTTCTGTTTTATCTAATCTGGCCACCTCCAAAAGTGGAAAGAAAGATCCAGAAGAT
             ************************************************************

M_413        GGGACCATGATGAGTTGCTCTATTGTTGATGGCAGCGAAAGCTATCAAGAGCAAACTTGT
Her_413      GGGACCATGATGAGTTGCTCTATTGTTGATGGCAGCGAAAGCTATCAAGAGCAAACTTGT
M_449        GGGACCATGATGAGTTGCTCTATTGTTGATGGCAGCGAAAGCTATCAAGAGCAAACTTGG
F_449        GGGACCATGATGAGTTGCTCTATTGTTGATGGCAGCGAAAGCTATCAAGAGCAAACTTGT
Her_449      GGGACCATGATGAGTTGCTCTATTGTTGATGGCAGCGAAAGCTATCAAGAGCAAACTTGT
             *********************************************************** 

M_413        TTTCATCAAGGTACCAGTTGTTTTGTCCTTGCTATACTGTTTATGATTGTAGGGGCATGA
Her_413      TTTCATCAAGGTACCAGTTGTTTTGTCCTTGCTATACTGTTTATGATTGTAGGGGCATGA
M_449        TTTCATCAAGGTAACAGTTGTTTTTTCCTGGCTGTACTGTTTATGATTGTAGGGGCATGA
F_449        TTTCATCAAGGTAACAGTTGTTTTTTCCTGGCTGTACTGTTTATGATTGTAGGGGCATGA
Her_449      TTTCATCAAGGTAACAGTTGTTTTTTCCTGGCTGTACTGTTTATGATTGTAGGGGCATGA
             ************* ********** **** *** **************************

M_413        CATTCTAATTCAAAGTCTGTGGTTTTACAAAGTTAAACATCTGCTATATTCTTGTGCAGG
Her_413      CATTCTAATTCAAAGTCTGTGGTTTTACAAAGTTAAACATCTGCTATATTCTTGTGCAGG
M_449        CATTCTAATTCAAAGTCTCTGGTTTTACAAAGTTAAACATCTGCTATATTCTTAGTCATT
F_449        CATTCTAATTCAAAGTCTCTGGTTTTACAAAGTTAAACATCTGCTATATTCTTAGTCATT
Her_449      CATTCTAATTCAAAGTCTCTGGTTTTACAAAGTTAAACATCTGCTATACTCTTAGTCATT
             ****************** ***************************** ****   **  

M_413        ------------------------------------GCTCAATGAGAGCTATGGTGACGT
Her_413      ------------------------------------GCTCAATGAGAGTTATGGTGACGT
M_449        ATGATTTAGCTTTTCTGACCTGGTCCCTTGTGCAGGGCTCAATGAGAGTTATGGTGACGT
F_449        ATGATTTAGCTTTTCTGATCTGGTCCCTTGTGCAGGGCTCAATGAGAGTTATGGTGACGT
Her_449      ATGATTTAGCTTTTCTGATCTGGTCCCTTGTGCAGGGCTCAATGAGAGTTATGGTGACGT
                                                 ************ ***********

M_413        CAGGAGTAATAATCTGCTCACCAAGAA
Her_413      CAGGAGTAATAATCTGCTCACCAAGAA
M_449        CAGGAGTAATAATCTGCTCACCAAGAA
F_449        CAGGAGTAATAATCTGCTCACCAAGAA
Her_449      CAGGAGTAATAATCTGCTCACCAAGAA
             ***************************
```

Supplementary Figure 1. Sequence of the *VviFSEX* gene in all three flower types. Sequence of the *VviFSEX* gene in *V. v. sylvestris* male (M), female (F) and hermaphrodite *V. v. vinifera* (Her) plants. M_449, F_449 and Her_449 represents the 449 bp fragment. M_413 and Her_413 represent the 413 bp fragment. In red, primers used for fragment amplification. Red boxe, region lacking 36 bp in M_413 and Her_413 sequences.

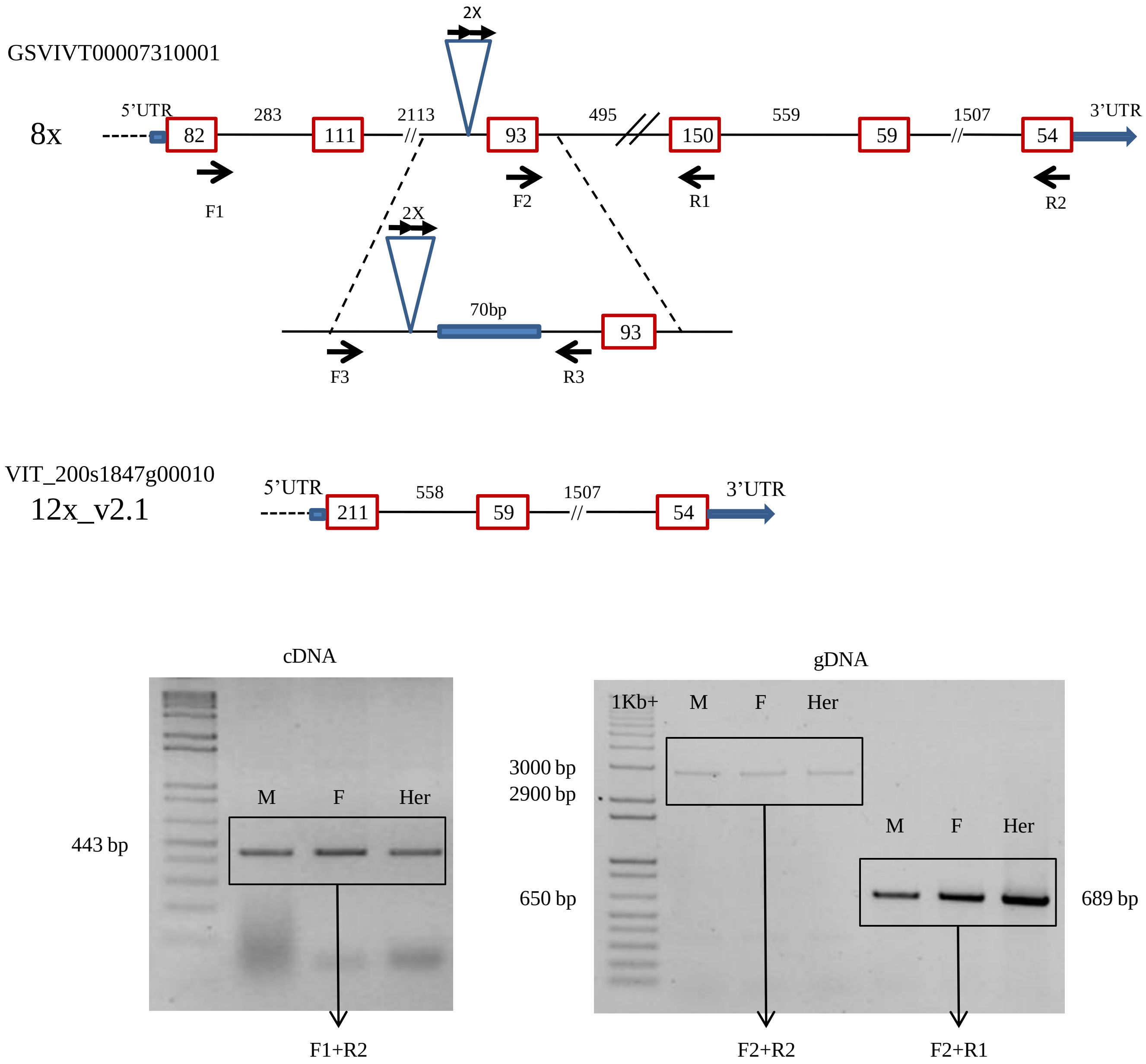

Supplementary Figure 2. *VviAPRT3* structure and amplification. *VviAPRT3* structure in the 8x and 12x_v2.1 annotation and gene amplification. Amplification with primers F1 + R2 in cDNA results in a fragment of 443 bp in concordance with the 8x version. Amplification with primers F2 + R2 and F2 + R1 in genomic DNA results in fragments with 2900 and 689 bp respectively. Amplification of *VviAPRT3* is in concordance with the 8x annotation version and not with the most recent 12x_v2.1 version. For primer sequences, see Fig. S1.

```
Her_541         TCTTTAGTATGAATGAATGTGCCTTCTAGACCCTGTTTCAGAGACTACTTCCAGACCCAG 60
M_541           TCTTTAGTATGAATGAATGTGCCTTCTAGACCCTGTTTCAGAGACTACTTCCAGACCCAG 60
F_542           TCTTTAGTATGAATGAATGTGCCTTCTAGACCCTGTTTCAGAGACTACTTCCAGACCCAG 60
M_611           TCTTTAGTATGAATGAATGTGCCTTCCAGACCATGTTTCAGAGACTACTTCCAGACCCAG 60
                ************************** ***** ***************************

Her_541         TT-CAGAGACTATTGTGAAATTTGATAGCATTTCCTTTATTGTAACTCTAGTGCTCACTT 119
M_541           TT-CAGAGACTATTGTGAAATTTGATAGCATTTCCTTTACTGTAACTCTAGTGCTCACTT 119
F_542           TT-CAGAGACTATTGTGAAATTTGATAGCATTTCCTTTATTGTAACTCTAGTGCTCACTT 119
M_611           TTTCAGAGACTATCTTAAAATTTGATAGCATTTCCTTTATTGTAACTCTAGTGCTCACTT 120
                ** **********  * ********************** ********************

Her_541         TTCCTTGTGAAAAATATTCTGTATCCGTATTCTTGACAAATTGTTGCTTACAGTTGCAAC 179
M_541           TTCCTTGTGAAAAATATTCTGTATCCGTATTCTTGACAAATTGTTGCTTACAGTTGCAAC 179
F_542           TTCCTTGTGAAAAATATTCTGTATCCGTATTCTTGACAAATTGTTGCTTACAGTTGCAAC 179
M_611           TTCCTTGTGAAAAATATTCTGTATCCGTATTCTTGACAAAATGTTGCTTACAGTTGCAAC 180
                **************************************** *******************

Her_541         TTTTTCCATTTACTGCTGATTCTTTGTCAAAATGTTCTGTATCCGTATTCTTGACAATTG 239
M_541           TTTTTCCATTTACTGCTGATTCTTTGTCAAAATGTTCTGTATCCGTATTCTTGACAATTG 239
F_542           TTTTTCCATTTACTGCTGATTCTTTGTCAAAATGTTCTGTATCCGTATTCTTGACAATTG 239
M_611           TTTTTCCATTTACTGCCAATTCTTTGTCAAAATGTTCTGTATCCATATTCTTGACAATTG 240
                ****************  ************************** ***************

Her_541         TGTAAACTTGACTAAGATCTCTGCATCCTGTATCCGTATTCTTGACAAAATGTTGCTTAC 299
M_541           TGTAAACTTGACTAAGATCTCTGCATCCTGTATCCGTATTCTTGACAAAATGTTGCTTAC 299
F_542           TGTAAACTTGACTAAGATCTCTGCATCCTGTATCCGTATTCTTGACAAAATGTTGCTTAC 299
M_611           TGTAAACTTGACTAAGATCTCTGCATCCTG-ATCCATAT-CTTGTCAAAATGTTGGTTAC 298
                ****************************** **** *** **** ********** ****

Her_541         AGTTGCAACTTTTTCCATTTACTGCT-ATTCTTTGACAAAATGTTCTGTATCTGTATTCT 358
M_541           AGTTGCAACTTTTTCCATTTACTGCT-ATTCTTTGACAAAATGTTCTGTACCTGTATTCT 358
F_542           AGTTGCAACTTTTTCCATTTACTGCT-ATTCTTTGACAAAATGTTCTGTATCTGTATTCT 358
M_611           AGTTGCAACTTTTTCCATTTGCTGCTGATTCTTTGACAAAATGTTCTGTATCTGTATTCT 358
                ******************** * *** *********************** *********

Her_541         TGACAATCGTGTAAACTTGACTAAGATCTT------------------------------ 388
M_541           TGACAATCGTGTAAACTTGACTAAGATCTT------------------------------ 388
F_542           TGACAATCGTGTAAACTTGACTAAGATCTT------------------------------ 388
M_611           TGACAATCATGTAAACTTGACTAAGATCTTTGCATCCTAAAAAATAGCTTCATACATTGA 418
                ******** *********************

Her_541         ----------------------------------------CACACTCAAG-CTGTTAGTT 407
M_541           ----------------------------------------CACACTCAAG-CTGTTAGTT 407
F_542           ----------------------------------------CACACTCAAG-CTGTTAGTT 407
M_611           GGCTTGGTCAAACAGGATTTTCTTTTATAGTTTTTATCTTCACACTCAAG-CTGTTAGTT 477
                                                         ********* *********

Her_541         AGGATTCTCGAAGTTCCTTAAATATTAAGA-CGTGACTTGTGATAGAATATCATTTTGGT 466
M_541           AGGATTCTCGAAGTTCCTTAAATATTAAGA-CGTGACTTGTGATAGAATATCATTTTGGT 466
F_542           AGGATTCTCGAAGTTCCTTAAATATTAAGATCGTGACTTGTGATAGAATATCATTTTGGT 467
M_611           AGGATTCTCGGAGTTCCTTAAATATTAAGA-TGTGACTTGTGATAGAATATCATTTTGGT 536
                ********** *******************  ****************************

Her_541         GAATTGGACCAAATTAAGGTGTTTAGCTCTTATTATATAGGATCATTTTTTCATTACTGA 526
M_541           GAATTGGACCAAATTAAGGTGTTTAGCTCTTATTATATAGGATCATTTTTTCATTACTGA 526
F_542           GAATTGGACCAAATTAAGGTGTTTAGCTCTTATTATATAGGATCATTTTTTCATTACTGA 527
M_611           GAATTGGACCAATTTAAGGTGTTTAGCTTTTATTATATAGTATCATTTTTTCGTTACTGA 596
                ************ *************** *********** *********** *******

Her_541         GGGAGGGCTGAGTTT 541
M_541           GGGAGGGCTGAGTTT 541
F_542           GGGAGGGCTGAGTTT 542
M_611           GGGAGGGCTGAGTTT 611
                ***************
```

Supplementary Figure 3. Sequence of the *VviAPRT3* gene in three *Vitis* flower types. Sequence of the second exon of *VviAPRT3* gene in *V. v. sylvestris* male (M), female (F) and hermaphrodite *V. v. vinifera* (Her) plants. M_611 represents the fragment of 611 bp. M_541, F_542 and Her_541 represent the fragment of 541 bp. In red, primers used for fragment amplification. Red box, region where male plants have 70 more bp than female and hermaphrodite plants.

Supplementary Table 1. Genes in the putative sex locus region of 143kb, located in the Chromosome 2 which display high identity with genes in the Chromosome Unknown. (*) genes absent in chromosome 2, 12x_v2.1 annotation genome but referred as located in this chromosome in several *Vitis* works present in NCBI database (http://www.ncbi.nlm.nih.gov/). Homology represents the similarity between genes from chromosome 2 and chromosome Unknown after and alignment between both sequences. (**) refereed here as *VviFSEX*.

Annotation	Chromosome 2		Chromosome Unknown	Homology
	Location (bp)	Code		
Trehalose-6-phosphate phosphatase	4897621..4903784	VIT_202s0154g00110	VIT_200s0233g00030	98%
Unknown	4914168..4914984	VIT_202s0154g00120	VIT_200s0233g00051	97%
Exostosin (Xyloglucan galactosyltransferase KATAMARI 1)	4921780..4923581	VIT_202s0154g00130	VIT_200s0233g00050	98%
3-oxoacyl-[acyl-carrier-protein] synthase 3 A, KASIII	4921664..4935576	VIT_202s0154g00140	VIT_200s0233g00060	98%
PLATZ transcription factor	4949210..4950534	VIT_202s0154g00150	VIT_200s0233g00100	96%
Ethylene overproducer like 1 (ETO1) *	-	-	VIT_200s0233g00090	-
Flavin-containing monooxygenase family	4951841..4956070	VIT_202s0154g00160	VIT_200s0233g00120	97%
Flavin-containing monooxygenase 3	4957715..4960913	VIT_202s0154g00170	VIT_200s0233g00130	79%
Flavin-containing monooxygenase 3	4962382..4965728	VIT_202s0154g00180	VIT_200s0233g00170	96%
Flavin-containing monooxygenase 3	4974431..4978234	VIT_202s0154g00190	VIT_200s0233g00180	97%
Unknown protein (*VviFSEX***)	4981728..4986851	VIT_202s0154g00200	VIT_200s0233g00190	99%
WRKY DNA-binding protein 21	4989461..4990306	VIT_202s0154g00210	VIT_200s2547g00010	99%
Adenine phosphoribosyltransferase APRT3 / APRT1 *	-	-	VIT_200s1847g00010	--
Unknown	5025223..5026178	VIT_202s0154g00220	-	-
Phosphatidic acid phosphatase / PAP2 *	-	-	VIT_212s0057g01540	-

Supplementary Table 2. Comparison of putative sex locus region of 143kb between sequence versions. Chr, chromosome; n.e., not existe; Unk, chromosome Unknown. (*) refereed here as *VviFSEX*.

Grapevine sex locus	8x (GENOSCOPE)			12x v0 (GENOSCOPE)			12x v2.1 (CRIBI)		
	Chr	Position	ID	Chr	Position	ID	Chr	Position	Annotation
Trehalose-6-phosphate phosphatase	2	5005678.. 5008670	GSVIVT00007296001	2	4898034..4901145	GSVIVT01001275001	2	4897621..4903784	VIT_202s0154g00110
Unknown	2	5056504.. 5057328	GSVIVT00007297001	2	4914168..4914984	GSVIVT01001276001	2	4914168..4914984	VIT_202s0154g00120
Exostosin (Xyloglucan galactosyltransferase KATAMARI 1)	2	5059842.. 5061675	GSVIVT00007298001	2	4921780..4923609	GSVIVT01001277001	2	4921780..4923581	VIT_202s0154g00130
3-oxoacyl-[acyl-carrier-protein] synthase 3 A, chloroplast precursor KASIII	2	5061793.. 5074025	GSVIVT00007299001	2	4923752..4935337	GSVIVT01001278001	2	4921664..4935576	VIT_202s0154g00140
PLATZ transcription factor (Blast TAIR)	2	5111107.. 5112422	GSVIVT00007302001	2	4948964..4950534	GSVIVT01001279001	2	4949210..4950534	VIT_202s0154g00150
Ethylene overproducer like 1 (ETO1)	2	5075581 .. 5095606	GSVIVT00007300001	2	4953998..4959471	GSVIVT01004781001	Unk	16074463..16074568	VIT_200s0233g00090
flavin-containing monooxygenase family	2	5114073.. 5157550	GSVIVT00007303001	2	4951821..4956004	GSVIVT01001280001	2	4951841..4956070	VIT_202s0154g00160
flavin-containing monooxygenase 3	n.e	n.e	n.e	2	4957741..4960852	GSVIVT01001281001	2	4957715..4960913	VIT_202s0154g00170
flavin-containing monooxygenase 3	2	5139881 .. 5151066	GSVIVT00007305001	2	4962612..4965728	GSVIVT01001282001	2	4962382..4965728	VIT_202s0154g00180
flavin-containing monooxygenase 3	2	5154260 .. 5157724	GSVIVT00007306001	2	4974657..4978139	GSVIVT01001284001	2	4974431..4978234	VIT_202s0154g00190
Unknown protein (*VviFSEX*)*	2	5161395 .. 5164520	GSVIVT00007307001	2	4983356..4986675	GSVIVT01001285001	2	4981728..4986851	VIT_202s0154g00200
WRKY DNA-binding protein 21	2	5172535.. 5172852	GSVIVT00007309001	2	4989461..4989778	GSVIVT01001286001	2	4989461..4990306	VIT_202s0154g00210
Adenine phosphoribosyltransferase APT3 / APT1	2	5189445.. 5196376	GSVIVT00007310001	Unk	40500627..40503367	GSVIVT01005518001	Unk	40500627..40503571	VIT_200s1847g00010
Unknown locus	2	5197902.. 5198867	GSVIVT00007311001	2	5025234..5026199	GSVIVT01001287001	2	5025223..5026178	VIT_202s0154g00220
Phosphatidic acid phosphatase / PAP2	UnK	43832801 .. 43840991	GSVIVT00007320001	12	10865289..10873477	scaffold_57	12	10292980..10293136	VIT_212s0057g01540
